# Evaluating Residential Segregation’s Relation to the Clustering of Poor Health across American Cities

**DOI:** 10.3390/ijerph17113910

**Published:** 2020-06-01

**Authors:** Joseph Gibbons, Tse-Chuan Yang, Elizabeth Brault, Michael Barton

**Affiliations:** 1 Department of Sociology, San Diego State University, San Diego, CA 92182, USA; 2 Department of Sociology, University at Albany, SUNY, Albany, NY 12222, USA; tyang3@albany.edu; 3 Department of Sociology, Louisiana State University, Baton Rouge, LA 70802, USA; emarks7@lsu.edu (E.B.); mbarto3@lsu.edu (M.B.)

**Keywords:** residential segregation, self-rated health, Moran’s I, poor health clustering

## Abstract

Residential segregation by race/ethnicity is widely recognized as a leading source of health disparities. Not clear from past research, however, is the overall health burden cities face due to clustering brought about by segregation. This study builds on previous research by directly measuring how spatially unequal health outcomes are within segregated cities. Utilizing Census-tract data from the Center for Disease Control and Prevention’s 500 Cities project, we examine how different dimensions of spatial segregation are associated with the clustering of poor self-rated health in cities. We make novel usage of the Global Moran’s I statistic to measure the spatial clustering of poor health within cities. We find spatial segregation is associated with poor health clustering, however the race/ethnicity and dimension of segregation matter. Our study contributes to existing research on segregation and health by unpacking the localized associations of residential segregation with poor health clustering in U.S. cities.

## 1. Introduction

Health disparities are an enduring global problem, but they are most apparent in cities suffering high racial/ethnic segregation where short distances can translate into vast disparities in health. For example, residents of New Orleans, United States who resided in the predominantly non-Hispanic White (henceforth White) areas two miles to the northeast from the city core lived on average for twenty five years longer than those in the predominantly non-Hispanic Black (henceforth Black) core [1]. (The racial component of this figure was determined by comparing Robert Woods Johnson Foundation maps to census tract-level 2011−2014 American Community Survey data created by authors). This kind of spatial disparity in poor health points to spatially concentrated, or clustered areas; poor health was spatially agglomerated in contrast with healthier parts of a city on a multi-neighborhood scale. Clusters of poor health indicate localized problems, like social disorder, that exacerbate existing health problems for those within these places [2,3,4,5]. These clusters also create serious issues for healthcare providers who struggle to manage concentrations of poor health in their respective cities [6].

Residential segregation is the physical manifestation of individual and systemic discrimination against people of color, which has unfolded over generations [7,8,9,10]. Over the 20th century, Blacks, and to a somewhat lesser degree Asians and Hispanics [11], have endured considerable individual discrimination from landlords and home sellers and systematic discrimination due to government sanctioned disinvestment in the form of redlining [12]. The problems of these neighborhoods have been exacerbated recently by predatory lending and the resulting 2008 housing crisis [13]. These concentrated disadvantages contributed to the development of structural barriers to people of color in the United States [14].

Segregation has long been linked with poor health [15,16,17,18,19,20] with the association initially identified by Yankauer in research on the relationship of infant mortality and residence in 1950 [21]. The association of racial/ethnic segregation with the local spatial clusters of poor health across cities remains less understood. First, most of the existing national-scale studies on racial/ethnic segregation and the spatial concentration of health disadvantage have focused on variation *between* cities, with little direct study of the clusters of poor health that exist *within* cities nationwide [22]. As such, it is difficult to determine how consistently racial/ethnic segregation relates with the clustering of poor health. Second, segregation has many different physical manifestations, or dimensions [23,24], each of which has unique implications for health [25,26]. Much of the existing research on health and segregation focused on only one dimension [26]. Third, the existing work on racial/ethnic segregation and health often used aspatial measures of segregation such as the dissimilarity index [24]. These kinds of approaches overlook the potential clustering or isolation of racial/ethnic groups which could, in turn, affect the spatial clustering of poor health [24,27,28]. Fourth, racial/ethnic segregation’s association with health clusters may vary depending on the racial/ethnic minority group segregated from Whites [16,26].

This study builds upon research on racial/ethnic segregation and health by assessing the association of spatial racial/ethnic segregation with the clustering of health problems. Data limitations prevented full examination of these questions until recently, but the Center for Disease Control and Prevention’s (CDC) 500 Cities project offers non-restricted Census-tract level health estimates for the 500 largest cities in the United States [29]. The current study takes advantage of the 500 Cities data to evaluate the association of racial/ethnic segregation with poor self-rated physical health *within* and *between* cities across the United States (U.S.). To understand the scope of racial/ethnic segregation’s association with the clustering of health problems, we adopt a measure of spatial clustering based on the Moran’s I statistic to investigate whether spatial racial/ethnic segregation relates to spatial health disparities in U.S. cities. While we cannot test the relationship of racial/ethnic segregation with the health of specific racial/ethnic groups, this novel dataset allows us to broadly examine racial/ethnic segregation’s relation with health disparities.

### 1.1. Social Sources of Poor Health Clusters

The presence of clusters of poor health within cities are strongly associated with local social determinants such as particular socio-economic disadvantage [30,31]. Research frequently demonstrated social disadvantage clusters within and between specific neighborhoods and frequently builds upon itself [32,33]. A key implication of the concentration of high poverty is the loss of social stability and cohesion in a neighborhood because residents do not spend enough time in their neighborhood to develop local social ties [33].

Social disadvantage can lead to the clustering of poor health in at least four ways. First, low income and unstable neighborhoods can be the site of many social stressors, like high crime, which can be detrimental to health [34,35]. Second, the breakdown of social order in a community can compound into the emergence of poor health behaviors such as the resistance to get vaccinations [36], failing to engage in timely cancer screening [37,38], or not practicing safe sex [39]. Third, low income communities often lack concentrations of local resources like quality food [40] or healthcare [30]. Fourth, low income communities are more likely to be vulnerable to environmental issues ranging from close proximity to highways [41] to concentrations of older housing stock that contains lead paint or other housing deficiencies that negatively influence health [42].

### 1.2. Spatial Character of Racial/Ethnic Segregation

There is some debate about whether racial/ethnic segregation or economic inequalities were more strongly associated with poor health [16,20,43,44]. While these two effects can be difficult to untangle across cities on a nationwide scale [45], there is some cause to suspect racial/ethnic segregation has a more fundamental effect on health clustering. Racial/ethnic segregation has a unique role in the formation of disadvantage and by extension poor health clusters. The residents confined to these neighborhoods are uniquely at risk to health problems. Segregated non-White communities are associated with political alienation and powerlessness and are consequentially at unique risk to health dangers [20] -- either directly, through the construction of pollutants in their neighborhoods like trash incinerators [46] and lead emissions [47], and indirectly, through inconsistent policing [32,33] or lack of oversight of safety in older housing stock [42,48]. This neglect also contributes to the vulnerability of these communities to illness, such as the failure of the government and public health system to intervene on HIV in the 1980′s and 90′s [39]. They are less likely to have the resources needed to manage these problems, such as quality healthcare, due to the powerlessness [49].

While segregation is an inherently spatial process, it was frequently operationalized in an aspatial fashion [24]. Extant research that incorporated aspatial segregation measures found the physical character of separation between ethnic groups was an important predictor of health outcomes [25,26]. These spatial patterns include the evenness of distribution of different racial/ethnic groups across a city or metropolitan area and how isolated racial/ethnic groups are from one another, isolation [23,24,27]. According to Grady and Darden [8], the uneven distribution of minorities indicates that across all neighborhoods in a city, some neighborhoods host more minorities than the city-level composition while others have fewer minority residents. According to Census 2010, the Detroit-Livonia-Dearborn metropolitan area observed the highest level of uneven distribution between blacks and whites in the U.S. [50]. Isolation suggests that minorities are more likely to be exposed to their co-ethnics than whites. For example, Hispanics in the Laredo metropolitan in Texas are the most isolated in contrast to Hispanics living in other metropolitan areas [50]. Consequently, not all dimensions of racial/ethnic segregation share the same spatial implication. A very segregated city could be characterized by a minority population unevenly distributed into one large section of the city or isolated across the city in pockets.

The divergent spatial character of segregation has implications for where disadvantage concentrates and affects local health. As such, different dimensions of racial/ethnic segregation may distinctly relate to health outcomes [26]. Given their shared spatial character, we hypothesize racial/ethnic segregation based on the uneven distribution of non-White neighborhoods would be more directly related to clustered poor health than racial/ethnic segregation based on isolation. Isolation, in contrast, can lead to an even, albeit isolated, distribution of minorities across space [17,43]. For example, a large concentration of a minority population would be more likely to be exposed to concentrated environmental hazards, such as local pollutants, than isolated minority communities surrounded by White neighborhoods which would feel this pollution’s effect as well [41,51]. Additionally, a very unevenly distributed minority population makes it easy to channel resources to White neighborhoods, thereby limiting access to health care and hence exacerbating the clustering of poor health [52]. For isolation, resources may be more evenly distributed as it is relatively difficult to separate minorities from the majority group.

Though the uneven distribution of racial/ethnic minorities is expected to impose a stronger impact on the clustering of poor health than isolation, it does not mean isolation is irrelevant. We argue that isolation may be subtly related with the local concentration of poor health. For example, isolation was found to have similar effects on the spread of disease clustering to uneven racial/ethnic populations [15]. Also, isolated communities often feature other forms of disadvantage which could relate to the spatial clustering of poor health, such as joblessness and crime [7,32,53]. Further, a minority community with low chance of exposure to the majority population would be more likely to experience a sense of isolation and powerlessness, which in turn could lead to a concentration of health problems [54,55]. Despite these possibilities, no study has directly compared the relationship of different spatial segregation measures with the clustering of poor health.

### 1.3. Racial/Ethnic Segregation’s Health Advantage?

While segregation was commonly associated with poor health, there remains a disparate body of work that highlights how residence in a minority community can offer some protective effects, especially among foreign-born non-Black minorities, namely Hispanic and Asian communities [56,57]. This has been frequently described as an ‘enclave effect’ [58,59] and has been framed as an ‘ethnic density effect’ previously in the literature [60,61,62,63]. While enclaves can include shares of large foreign born, this depends on the ethnic group and their location [60,62,64]. Examples of this protective effect include the ‘Latino Health Paradox,’ a way to describe the longer life spans of some first-generation Hispanic immigrants even when accounting for socio-economic disadvantage [65].

This enclave effect takes on several characteristics. For one, minorities living in mostly non-White communities were shielded from the direct effects of racial/ethnic discrimination [61]. Another benefit of the enclave effect is that minorities were more likely to form social ties in these communities than they would in predominately White communities [66]. Local friendship and family networks have been found to offset poor health for the Latino community, helping to explain the Latino health paradox [65]. Stronger ties increase the likelihood community members will look out for one another in times of trouble such as during health crises [67] or share information on where to get care [68]. Communities with large foreign-born populations can also benefit from transnational social ties which can provide added resources compared to native born non-Whites [53,69]. Indeed, one common explanation for racial/ethnic segregation is that minorities intentionally selected these neighborhoods for the above benefits [10,69], though the endurance of these benefits over time is questionable [70]. Lastly, concentrations of ethnic minority groups can lead to more culturally sensitive healthcare services and healthcare providers that speak minority languages [71].

At present, the evidence of protective effects from racial/ethnic segregation for non-Black minority communities remains inconclusive. Sampson [33] reported socio-economic disadvantage found in segregated communities inhibited the development of strong community ties. Indeed, highly disadvantaged Hispanic communities were found to have trouble maintaining social ties over time [72]. Meanwhile, though Asian communities vary greatly by nationality and race, they were less likely to experience the kind of economic disadvantage found in predominately Black and Hispanic neighborhoods [69]. It is also unclear how exclusive the enclave effect was for non-Black minorities compared to Blacks [64]. For instance, Black communities are known to contain health promoting social networks [37] that can also shield their residents from discrimination [73]. On the other hand, Black communities are argued not to have the same degree of health-based social support as immigrant non-Black communities [74]. In addition, Blacks have endured a unique discrimination from healthcare providers which has led to widescale distrust of providers [74]. Indeed, Black and Hispanic residents often avoid healthcare because of the concern that they will be treated differently [75]. Gibbons and Yang [16] find worse health outcomes for Blacks living in White areas, but they did not find health benefits of being Black and living in a mostly Black community. Lastly, these strong communities may discourage healthy behaviors. For example, there is evidence of both Asian and Latino communities discouraging their peers to seek out cancer screening due to community distrust of these practices [76].

### 1.4. Hypotheses

The spatial character of racial/ethnic segregation within cities was often associated with the concentration of disadvantage [9,33]. We suspect that the concentration of disadvantage relates to the concentration of health problems. To explore this dynamic, we test the following hypotheses. Regarding the association of racial/ethnic segregation with the clustering of poor health in U.S. cities when controlling for factors like city SES, we expect:

**Hypothesis** **1.** Controlling for other relevant characteristics, White/Black residential segregation was positively related to the clustering of poor health because minorities are more likely to live in disadvantaged areas where exposure to health risks is higher and access to health care is lower.

We also suspect that even when controlling for SES non-Black minority segregation from Whites had less of a relationship with the clustering of poor health than Black segregation from Whites due to the enclave effect [56,57,59]:

**Hypothesis** **2.** Controlling for other relevant characteristics, White/Hispanic residential segregation is negatively related to the clustering of poor health because greater exposure to health risks is often offset by advantages associated with living in ethnic enclaves.

**Hypothesis** **3.** Controlling for other relevant characteristics, White/Asian residential segregation is negatively related to the clustering of poor health because Asians are more likely to live in ethnic enclaves that provide access to resources associated with improved health.

Based on the existing understanding of the dimensions of racial/ethnic segregation and health [25,26], we believe that even when controlling for SES the uneven spatial distribution of non-Whites is more related with the clustering of poor health than the exposure of non-Whites to Whites:

**Hypothesis** **4.** Based on the characteristics of both dimensions of spatial segregation, we suspect the measure of spatial evenness had a stronger association with the clustering of poor health than spatial isolation.

## 2. Materials and Methods

### 2.1. Dependent Variables

Our dependent variables were derived from the 500 Cities project, which created Census tract-level estimates of the 2014 wave of the Behavioral Risk Factor Surveillance System (BRFSS) for 500 American cities. The BRFSS is a national household telephone survey administered every two years by the CDC with identifiers down to the county level. Tract estimates from the BRFSS were derived through a multilevel strategy linking geocoded county-level BRFSS data to block-level demographic data from the 2010 Census, including age, sex, and race/ethnicity, to predict the characteristics of health by location [29,77,78,79]. The CDC primarily selected Census designated places with the ‘city’ designation and at least 66,000 residents as of 2010 for these estimates (the CDC makes a few exceptions to this strategy to ensure at least one city for each state. This includes Honolulu, HI, which is not technically designated as a city. Also, they include several cities with populations below 60,000 in 2010). The 500 Cities estimates were validated through two methods: (1) comparing the city-level estimates with existing corresponding BRFSS data; (2) comparing the tract-level estimates with local BRFSS results in Boston, MA [77,79]. Nonetheless, there is some risk that the BRFSS estimates will unexpectedly correlate with the other demographic data in the models. Some caution should be had in interpreting our findings. While this data was available at the tract level, our analyses are at the city level because we are interested in examining variation across cities.

The measure *poor health clustering* is derived from the 500 Cities measure “Poor self-rated physical health” which indicates the percent of residents in tracts “…aged ≥18 years who report 14 or more days during the past 30 days during which their physical health was not good” [80]. Self-rated health was chosen because it not only correlates strongly with ‘more objective’ measures like mortality [2,81], but also because it enables us to identify people feeling of poor health even if their issue could not be identified with an objective measure [82]. Thus, while self-rated health may not be as precise as other measures, it is an inclusive way to establish the general health of a neighborhood. While this variable is a variant of the conventional self-rated health indicator (i.e., a Likert scale) used in surveys [2,16], it is an appropriate assessment of population health at the community/neighborhood level. Several authors of this study have used this dataset to examine percent self-rated health by census tracts in a city. While they encountered many of the above issues, they found the data serving as a reasonable proxy of wellbeing [83].

We assess the spatial variation in poor self-rated physical health within cities by examining the clustering of poor self-rated health among census tracts for each city. Using clusters allows us to roughly measure the magnitude of spatial health disparity within a city. To identify these clusters, we make novel application of the Global Moran’s I statistic onto our self-rated physical health measures. Moran’s I is a method to identify the spatial autocorrelation of a variable. Spatial autocorrelation reflects the extent to which values of a neighborhood characteristic such as presence of poor self-rated health is predicted by adjacent neighborhoods [33,84]. This approach is based on characteristics being shared by neighborhoods, which means it miss out on lone places with unusually high or low values relative to their surrounding areas. Nonetheless, using a Moran’s I to assess poor health clustering is useful for three reasons.

First, the Moran’s I score is a commonly used measure of health and disease clustering, and thus using this allows better integration with existing research [28,85,86]. Second, it allows users to determine if the spatial clustering of unhealthy neighborhoods is statistically significant [87]. Establishing the significance of clusters ensures they are not due to other random effects. It goes beyond a simple descriptive indicator of how a phenomenon distributes across a region (such as entropy) [86]. A strong and significant Moran’s I score suggests city health outcomes are spatially unequal. Third, it considers the spatial relationships among neighborhoods when investigating health disparities across space. A conventional approach to geographic health concentration is to use the pre-defined feature of an area (e.g., population size/urbanicity) and how these spatial units are related is largely ignored. Fourth, autocorrelation suggests underlying local effects that are leading to the clusters.

A low or non-significant Moran’s I score means there are no measurable clusters of poor health that exist at a census tract level in a given city. This does not mean these cities have no neighborhood health problems, indeed, there could just be smaller pockets of poor health. However, the lack of these clusters infers that the health of a city is generally more evenly distributed spatially compared to cities with high and significant scores.

While there are other spatial clustering measures, the Moran’s I has several advantages over them [88]. For example, while Geary’s C [88] adopts the similar cross-product approach to assess spatial clustering, the values range between zero and two, making the interpretations less intuitive than the Moran’s I. Alternatively, the Getis-Ord G [89] distinguishes the clusters of low values from those of high values and indicates the dominant types of clustering in a study area, rather than the level of spatial clustering. A non-significant G value may indicate the equal presence of both clusters of low and high values without clear distinction between them. The Moran’s I instead captures both spatial similarity (positive values) and dissimilarity (negative values). It is important for this study to reflect dissimilarity because it corresponds to the isolation dimension of racial/ethnic segregation. Specifically, the Moran’s I can capture unhealthy communities surrounded by healthy communities. Without this feature, it may be difficult to understand “how” isolation is related to divergent spatial health disparities. As such, the Moran’s I serves the purpose of this study best.

To derive the Moran’s I scores, spatial weights matrices were created for the Census tracts of each city using the R package *spdep*, using a first-order queen continuity matrix (we also explored using a K-nearest neighbor (KNN) weighting strategy, but sensitivity analyses (available upon request) reveal that the KNN approach produced similar findings and conclusions). This weighting system accounts for all neighbors that directly share a border with a tract. The Moran’s I score for each city was computed based on these weight matrices. The resulting score is the correlation of self-rated physical health by neighborhood to its neighboring influences for a city overall, indicating clustering of health within a city’s neighborhoods [87]. Stronger correlations indicate more pronounced clustering of poor self-rated physical health among tracts within a city. We code non-significant Moran’s I scores as zero. These non-significant scores accounted for 22 percent of the sample (sensitivity analyses were also conducted with the non-significant values included. These results were largely consistent with the results reported. This supplemental analysis is available upon request).

There were several challenges in creating weights matrices for the sampled cities. First, several cities contained “islands, which were tracts that did not share boundaries with other tracts in the city due to the presence of features such as water reservoirs which can be miles from the rest of a city [90]. Such islands were omitted from our analyses. Second, the boundaries of census tracts for some cities do not conform to municipal boundaries, which meant some tracts were shared by two cities. These tracts were most often found in the south and west where municipal borders are generally more in flux. To address this, GIS software was used to determine whether a large majority, at least 60 percent, of a tract’s land area was present in its respective city. If it was not, the tract was omitted. Census tracts shared among cities were omitted from the study outright. The combination of island, outsider, and shared tracts constituted less than 2 percent of the tracts in the 500 Cities data. Another issue was that census tracts that are disproportionately larger than most tracts in a city can affect a weights matrix. Many of these large tracts were on the periphery of cities and were often omitted as one of the problem tracts listed before. Also, we omitted Honolulu, HI and Las Cruces, NM from our sample because we were unable to create a spatial weights matrix for those cities.

### 2.2. Spatial Racial/Ethnic Segregation Measures

Given the spatial character of our outcome, we chose to use two measures of spatial (instead of aspatial) segregation recommended by Reardon and O’Sullivan [24] as our focal predictors. Specifically, the current study uses the Spatial Information Theory index (H) for spatial evenness, and the Spatial Isolation Index (P*) for spatial exposure. The key difference between spatial and aspatial segregation measures is whether an indicator considers the spatial arrangement of population. Most of the conventional segregation measures, such as the dissimilarity index, are aspatial [24], which mismatches with our spatial poor health clustering measure. We follow the suggestions of existing research to concentrate on the evenness and exposure dimension of segregation [8,17,47]. H can be understood as a measure of how high residential segregation is between two groups, 1 indicating maximum segregation and 0 representing complete integration. While P* generates both spatial exposure and spatial isolation components, we focus on the latter in light of its well-documented effect on health [7,27]. P* can be interpreted as the probability of two randomly selected individuals being racial/ethnic minorities. For simplicity when discussing P*, we reference the Spatial Isolation Index in place of ‘exposure.’

More importantly, the following features make H and P* outperform other commonly used spatial segregation measures [24]. First, both H and P* can be decomposed with the change in the boundaries of subareas and the decomposed values are additive. Second, H and P* can be applied to both aggregated population counts (zone-based) or continuous population density (surface-based). The latter helps to minimize the well-known modifiable area unit problem [91,92]. For a fuller visualization of the spatial character of isolation and evenness, we recommend Iceland et al. [93].

Data for spatial residential segregation were collected from the 2010−2014 American Community Survey (ACS). The measures were calculated with Census tracts with the ‘*seg*’ package in R for each of the 498 cities separately [92] (as a reminder, Honolulu and Las Cruces, New Mexico were excluded from the analyses). While the option existed to conduct a multigroup measure, we chose to conduct our analysis on just two groups at a time, Blacks and Whites, Hispanics and Whites, and Asians and Whites for two reasons: First, two group measures are more readily interpretable than multi-group measures. Second, the two group measure places primacy on segregation from Whites as all the measures directly compare Whites from non-Whites. This is important given how much of an effect the separation from Whites is thought to have on non-White health disparities [17].

### 2.3. Other Independent Predictors

Our analyses also use the ACS to control for other relevant city-level predictors. Following the ethnic density framework [60,62,64], we account for potential foreign advantage with a measure of proportion foreign born. We include two measures of economic inequality. First, we measure overall income inequality in a city, based on the incomes of tracts, with the Gini coefficient. This measure allows us to determine if segregation’s influence on health clustering exists independent of income inequality [16,20,43,44]. Next, we include a measure of city socio-economic status (SES) based on factor analysis of the following components: percent unemployed (loading 0.629), percent in poverty (loading 0.995), a logged version of median household income (loading −0.898), and percent of those with no High School (loading 0.737). The resulting SES variable accounted for over 70 percent of the variation in these variables. In addition, we include measures of the proportion of female headed households in a city and median age of a city, given the associations these two factors have with health [94]. Our measure of residential stability includes the proportion of residents who have lived in the same housing for at least five years and proportion of home-owners [95]. We also control for city level population features by including a measure of the log transformed population count for each city. Given previous research that suggested regional variation, we included dichotomous measures of the region wherein a city is located (South, Midwest, West, and Northeast as the reference) [96]. Finally, we indicate whether each city is a primary city of a CBSA to control for unique health disadvantages in the core cities of a metropolitan area.

### 2.4. Methods

In conducting our analysis, we complete the following steps: First, we conduct simple descriptive analyses of the 498 cities in our sample. Second, to better contextualize the nature of our dependent variable, we complete Exploratory Spatial Data Analysis (ESDA). While our main analyses examine variation among cities, our ESDA examine tract level variation within cities. We use Local Indicators of Spatial Autocorrelation (LISA) analysis to assess the underlying character of the Moran’s I clusters at the tract level. While the LISA is a distinct analysis from the Global Moran’s I score, it utilizes a local iteration of the Moran’s I and in so doing identifies the clusters in the global scores. This approach identified statistically significant clusters of tracts within each of the 498 cities that featured high rates of poor self-rated health (High-High, HH) and statistically significant clusters of tracts within each of the 498 cities that featured low rates of poor self-rated health (Low-Low, LL) [87]. Also, LISA identifies outlier tracts, places with high concentrations of poor health directly adjacent to those with little or no concentration (High-Low) and vice versa (Low-High). Third, to directly examine the relationship of spatial segregation with spatial health clusters across the 498 sampled cities, we conducted OLS regression analyses.

To avoid confusion, we would like to emphasize that when creating the segregation indices and Moran’s I values, we focus on the within-city spatial variation and the census tracts within each city serve as the analytic unit in the process of variable creation. These segregation indices and Moran’s I values, in turn, reflect a city’s spatial characteristics and each city serves as the analytic unit in our regression analysis.

## 3. Results

### 3.1. Descriptive Results

The descriptive statistics for the sample of 498 cities included in our multivariate analyses are displayed in Table 1. The average city featured a poor health clustering score of 0.37, indicating moderate clustering. Our analyses measure spatial racial/ethnic segregation in two ways: the Spatial Information Theory index (H) and the Spatial Isolation Index (P*). While the average White/Black H value (0.18) is higher than the White/Hispanic (0.13) and White-Asian H (0.12) values, these measures display similar variation across our sample cities; the standard deviation of White/Black Spatial Information index is 0.12 while White/Hispanic and White-Asian indices have a standard deviation of 0.07 and 0.08, respectively. Compared to the Spatial Information Theory Index, the Spatial Isolation Index shows more variation across cities: all three—Asian/White, Black/White and Hispanic/White—have standard deviations near their means and ranges near both extremes. For example, the Asian/White isolation index has a mean of 0.19, a standard deviation of 0.17 and ranges from 0.02 to 0.85.

For ease in interpretation, we report the following proportions as percentages. The cities in our sample comprise an average of 17.3 percent foreign born, 41 percent female headed households, and an average median age of 35.71. On average, 57 percent of residents owned their homes and 29 percent lived in their home for at least five years. The socioeconomic status factor scores are right skewed with a maximum (3.28) 1.4 standard deviations away from the mean than the minimum (−1.88). Roughly 30 percent of the cities were in the South, 40 percent in the West, and 20 percent in the Midwest. The Northeast is the reference category and occupies the remaining ten percent. The average population of the sampled cities is just over 225,000, though the sample includes cities ranging in size from Burlington VT (42,342) to New York City (8,341,152). Many of the cities constitute the core of their respective metropolitan area, 68 percent of the sample are Primary Statistical Areas.

### 3.2. Exploratory Spatial Data Analysis

**Figure 1:**
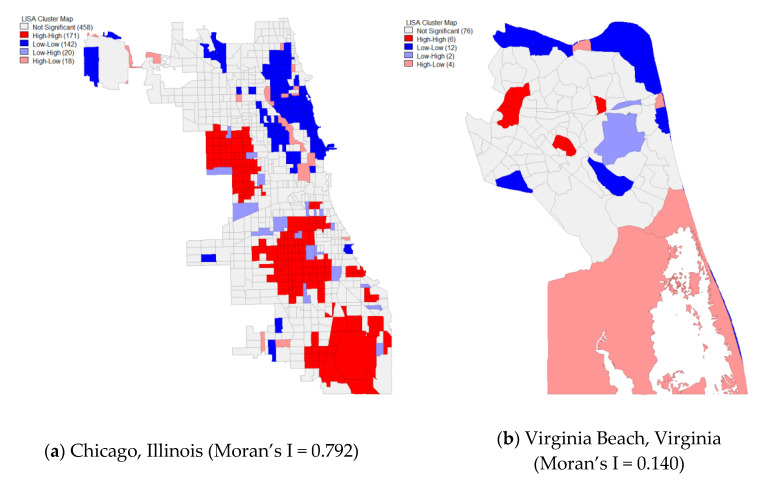
LISA Map of Rates of Poor Self-Rated Health for Two Cities.

We select two cities from our sample for LISA to visualize these clusters, one closest to the top quartile of the Global Moran’s Scores and one closest to the bottom quartile of the Global Moran’s score, to provide a reader a sense of how these clusters can appear within cities. The city with the higher score is Chicago, IL (Figure 1) with a Global Moran’s I of 0.792, meaning poor self-rated health is highly clustered in that place. The city with the lowest significant score is Virginia Beach, VA, which had a score of 0.140, meaning poor self-rated health was not very clustered in that city. The LISA map for Chicago shows stark disparities, with large clusters of HH and LL clusters occupying large sections of the city. In contrast, the clusters of HH and LL in Virginia Beach were more scattered across the city, with a less obvious pattern of systemic disparity compared to what was seen in Chicago.

It is important to stress these scores do not indicate Chicago was a dramatically less healthy city than Virginia Beach. Indeed, while 12.8 percent of Chicago residents reported poor health, 9.6 percent of Virginia Beach residents reported poor health, a difference of only 3.2 percent. Instead, the Moran’s I show that most of the people who reported poor health in Chicago were spatially concentrated while those reported poor health in Virginia Beach were more spread across the city. In short, clustering suggests that health outcomes were more unevenly distributed within a city. Further, these differences suggest a difference in residential racial/ethnic H or P* segregation rates between Chicago and Virginia Beach. Chicago has a longstanding reputation as one of the most segregated cities in the United States [9,10,33]. For example, the Black/White H for Chicago is 0.691, compared to Virginia Beach whose score is 0.152.

To gain a more thorough understanding of what the neighborhoods in the LISA clusters look like, we extract the tract level demographic data for all the cities in the sample and conduct descriptive statistical analysis by cluster tracts. The findings are summarized into Table 2. As expected the HH tracts were less healthy than other tracts, with an average 18.62 percent of residents in these areas reporting poor self-rated physical health compared to 13.03 percent in tracts overall and 8.69 percent in LL tracts.

Next, the HH tracts had higher shares of Black and Latino populations compared both to the tracts overall and LL tracts. For example, the percent Black in HH tracts is 35.21, compared to 20.44 percent in tracts overall and only 8.54 percent of LL tracts. Meanwhile, the LL tracts had larger shares of White populations and Asian populations than both HH tracts and tracts overall. Whites account for 67.26 percent of LL tracts, only 22.91 percent of HH tracts, and 45.75 percent of tracts overall. In addition, the High-High tracts had above mean poor SES (2.032), compared to the below mean scores of SES found in Low-Low tracts (−1.61) (the tract-level version of the SES variable was constructed with the same variables for the city-level SES measure. The loadings, available upon request, were very similar to their city-level counterpart). However, it should be noted that there is considerable variation in these values with almost all the standard deviations exceeding their respective means. *t*-Tests show almost all the cluster means are significantly different from the overall means. These results strongly indicate that poor health clusters identified with the Global Moran’s I scores are related to race/ethnicity and class. However, these results do not unequivocally confirm a relationship between residential H or P* segregation and racial/ethnic poor health concentrations. In the following sections, we directly explore the relation of segregation to poor health clustering by looking at the clusters overall at a city level.

### 3.3. Multivariate Results

The results of our OLS analyses of city level variation are reported in Table 3. We assess separate models for each dimension of spatial segregation by the race/ethnicity being measured because of the high collinearity attributed to measuring multiple dimensions of segregation in one model. Post-regression analysis of the models noted acceptable VIF scores below 5 for the remaining predictors used. Comparisons of coefficients across models were assessed using the technique described by Clogg, Petkova, and Haritou [97].

Foremost, the Spatial Information Theory Index score for Hispanics compared with Whites has a significant and positive relationship with poor health clustering. Each point of the Information Theory Index for Hispanics to Whites increases the Moran’s I for poor health by 0.331 points. Meanwhile, for White/Black and White/Asian evenness there is no significant relationship to poor health clustering. In other words, the uneven distribution of Blacks or Asians to Whites is not related to clustering of poor health. It should be noted, however, that the Black evenness measure is significant in models (not reported) where the Gini coefficient is not included, indicating an intertwined relationship between racial/ethnic segregation and income inequality among Blacks and Whites.

The Spatial Isolation Index reveals some unexpected trends. While the level of isolation of Blacks from Whites is not significant, the level of isolation Hispanics have from Whites has a significant positive relation with poor health clustering while the isolation of Asians from Whites has a significant *negative* relationship. With each point increase of isolation for Hispanics, the Moran’s I of poor health for a city increased by 0.150 points while each point of isolation for Asians decreased the poor health by 0.210 points. This suggests that isolation can have a relationship with the clustering of poor health. Model 5 is the only case where foreign-born is significant, carrying a negative relationship with poor health clustering. Supplemental models (not included) that did not control for percent foreign-born found Hispanic isolation is not significant. While our measure of foreign-born does not distinguish race and ethnicity, this suggests different health outcomes for Hispanics depending on their nativity status. Also, the spatial isolation of Asians can carry positive implications for the clustering of other characteristics like health.

Some of the other controls are significantly related with poor self-rated health. Most notably, the Gini coefficient is significant across all models – pointing to the importance of economic inequality and health. City population is also consistently significant, suggesting larger cities are more likely to have clustered poor health than smaller cities. Tracts located in cities in the Midwest are more likely to feature poor self-rated health, likely because of greater racial and economic inequality in the many rust-belt cities of this region [33,50]. Meanwhile, the SES measure is not significant, reaffirming the importance of segregation by race/ethnicity on poor health clustering. Location in a primary city is not significant in most models, suggesting that the disadvantage of core cities is less important than originally expected. It is difficult to say whether a suburban advantage/disadvantage exists given our lack of data on full metropolitan areas.

## 4. Conclusions

Residential segregation by race/ethnicity is a major suspect for the clustering of poor health in cities, but there has been no evidence of this local relationship nationwide. To better understand the relationship of poor health clustering and racial/ethnic segregation, we utilized the Moran’s I statistic to measure the spatial clustering of poor health within a selection of nearly 500 cities across the U.S. We examined the association of segregation with poor health clustering using two measures of spatial segregation, the Spatial Information Theory Index (H) and the Spatial Exposure Index (P*), referenced as the Spatial Isolation Index. Our study contributes to extant research on racial/ethnic segregation and health by unpacking the localized associations that segregation has with poor health clustering in U.S. cities.

We mixed support of an association between spatial evenness and poor health clustering. Our first hypothesis (H1), White/Black unevenness is associated with poor health clustering, was not supported. Alternatively, White/Hispanic unevenness is strongly associated with poor health clustering. This second association was not expected as we hypothesized (H2) White/Hispanic unevenness would have a negative relation with health outcomes due to the protective health effects of Hispanic communities.

Next, there is no association between White and Asian spatial unevenness. While this is not sufficient evidence to support our hypothesis (H3) that White/Asian segregation would have a measurably *negative* relation with health outcomes, we do not find an explicit positive relation with White/Asian unevenness and spatial health problems, either.

We found mixed support for the hypothesis (H4) that spatial isolation from Whites is not positively related to poor health clusters. While we find no relationship between Black and White isolation, we do find a positive relationship between Hispanic and White isolation and poor health clustering and a *negative* relationship between Asian isolation and poor health clustering. The former finding suggests that spatially isolated Hispanic communities indeed lead to the concentration of disadvantage, which in turn translates to poor health. This supports research about the importance of isolation for poor health [7,17], as well as the erosion of health advantage for some Hispanic populations between generations [70]. The Asian findings are aligned with two previous studies reporting a beneficial effect of isolation on health. Walton [56] and Mobley et al. [98] suggest that high levels of isolation lower the odds of low-birth-weight infants and heart disease, and a net of other covariates. Though other research has found evidence calling to question this trend [7,99]. Our finding extends their conclusions to the city-level. Clearly, spatial isolation can mean very different things depending on the ethnic group that is isolated.

The aggregate nature of our data limits our ability to explicitly say that non-Whites are the most affected by poor health clustering. We do report in Table 2 that Black and Hispanic populations constitute higher shares of residents in poor health clusters than elsewhere. Moreover, ample evidence from previous research demonstrates that non-Whites bear the brunt of poor health outcomes in segregated environments. As such, we confidently infer that non-Whites are experiencing the greatest health issues in cities with poor health clustering [7,16,17,26,51,56]. By extension, we also found in Table 2 that White populations had comparatively low representation in poor health clusters. Framed differently, our findings strongly suggest that White neighborhoods have more health advantage compared to non-White neighborhoods. However, this speculation will ultimately need to be verified through direct evidence.

Some of the associations of racial/ethnic segregation to health require further consideration. First, the lack of significance of Black/White evenness and health clusters was notable. However, it is worth consideration that when the Gini coefficient is not included in the model, White/Black unevenness is significant. This suggests that among Blacks and Whites, racial segregation, income inequality, and health problems are tangled [16,43]. However, segregation produces inequalities that contribute towards socio-economic inequalities and, by extension, the health inequalities [20]. Next, we did not find White/Hispanic evenness segregation had a negative relationship with poor health clustering as expected. This calls to question previous findings suggesting segregated non-Black minority communities have a health advantage [56,57,59]. We cannot directly single out the poor self-rated health of Hispanic residents in these places, but this suggests more research should be conducted about the Hispanic health paradox.

There is still some evidence of non-Black racial/ethnic advantage. The high exposure of Asians to other Asians may suggest an indirect relationship between the spatial concentration of Asian communities and better health. However, we do not have the disaggregated data to verify this assertion. In addition, the considerable variation among health effects for different Asian nationalities means that racial/ethnic segregation can have very different outcomes depending on the group [100]. Future work in this vein will need to unpack health differences for Asians by nationality.

While this study breaks important ground on the use of nationwide data on local health outcomes, there are several limitations within which future research must contend. First, the aggregated nature of the 500 Cities data raises the risk of ecological fallacy. While research on the association of racial/ethnic segregation and health has frequently analyzed multilevel models [17], much of this research was isolated to a single city. A related issue is the 500 Cities data does not disaggregate poor health among different racial/ethnic groups. Second, the cross-sectional nature of this study limited our ability to make causal assertions with our findings. Third, the 500 Cities excludes many suburban communities in metropolitan areas, which is problematic given segregation takes place on a metropolitan scale [9]. As such, we cannot say for certain this data assesses the full scale of racial/ethnic segregation’s effects onto individual health. Fourth, while we use the measures of spatial segregation considered to be the most ideal by Reardon and O’Sullivan [24], other measures might have yielded different findings [101]. While our dependent variable is not based on the conventional 5-level Likert scale [81], it is still likely that our dependent variable is subject to the systematic differential self-rated health assessment due to cultural or locational differences. Future research should investigate if the choice of self-rated health indicator matters.

Our findings suggest several policy recommendations. We agree with previous research that desegregation is the most direct way to address poor health clustering, but desegregation is a daunting task. Despite decades of policy efforts, racial/ethnic segregation is deeply rooted and reproduces itself through subtle discrimination as well as ingrained thinking during housing selection [10]. Partial desegregation is not sufficient to deal with poor health clustering as non-Whites living in mostly White communities still disproportionately report health problems [16]. A viable short-term solution to poor health clustering is for policy makers and public health officials to use Moran’s I scores to identify the most unequal cities and then utilize LISA maps to identify the clusters of poor health for targeted interventions. While the health inequalities of cities grow, there are methods to at least identify these problem areas and intervene. While this will not put a definitive end to health inequalities, it is a viable start.

## Figures and Tables

**Table 1:** Descriptive Statistics of Spatial Segregation Indices and Control Variables.

		Mean	Standard Deviation	Min	Max
Dependent Variable					
Poor Health Clustering (Queens Weighting)		0.37	0.21	0.00	0.83
Independent Variables					
Spatial Information Theory Index	White/Black	0.18	0.12	0.02	0.69
	White/Hispanic	0.13	0.08	0.01	0.43
	White/Asian	0.12	0.07	0.01	0.54
Spatial Isolation Index	Black/White	0.32	0.25	0.01	0.96
	Hispanic/White	0.39	0.25	0.04	0.98
	Asian/White	0.19	0.17	0.02	0.85
Gini Coefficient		0.21	0.05	0.08	0.38
Proportion Foreign-Born		0.17	0.11	0.01	0.72
Proportion Female Headed Households		0.41	0.20	0.34	0.47
Proportion Residing in Same Housing		0.29	0.05	0.14	0.50
Median Age		35.71	3.64	25.3	47.47
Proportion Home Owner		0.57	0.11	0.19	0.85
Socio-Economic Status		0.00	1.00	−1.88	3.28
Southern		0.31	0.46	0.00	1.00
Western		0.39	0.49	0.00	1.00
Midwestern		0.20	0.40	0.00	1.00
Total Population		225,222	475,118.9	42,342	8,341,152
Primary City		0.68	0.47	0.00	1.00
					N = 498

**Table 2:** Census Tract-Level Demographics by Self-Rated Physical Health Clusters.

	Overall		Low-Low Cluster		High-High Cluster	
Tract Statistics	Mean	St. Dev.	Mean	St. Dev.	Mean	St. Dev.
Proportion Poor Self-Rated Physical Health	0.13	0.04	0.08	0.02	0.18	0.03
Proportion Black	0.20	0.27	0.08	0.12	0.35	0.35
Proportion White	0.45	0.29	0.67	0.21	0.22	0.23
Proportion Asian	0.06	0.10	0.08	0.10	0.03	0.08
Proportion Hispanic	0.23	0.24	0.12	0.15	0.34	0.32
Proportion Foreign-Born	0.18	0.15	0.13	0.11	0.19	0.16
Proportion Female Headed Households	0.41	0.05	0.42	0.05	0.38	0.05
Proportion Residing in Same Housing	0.03	0.30	0.04 *NS*	0.49	0.03 *NS*	0.05
Proportion Home Owner	0.51	0.24	0.61	0.24	0.39	0.18
Socio-Economic Status	0.00	1.69	−1.61	0.93	2.03	1.54
Tract Population	4246.23	2100.47	4551.12	2515.77	3672.88	1722.78
Number of Tracts	29305		3519		3690	

Notes: All *t*-Tests Comparing cluster means to overall means reported significance (*p* < 0.001) unless noted (NS).

**Table 3:** Spatial Segregation and Poor Health Clustering for Cities - Queens Weighting. N = 498.

	Spatial Information Theory Index			Spatial Isolation Index		
	Model 1	Model 2	Model 3	Model 4	Model 5	Model 6
	White Black	White Hispanic	White Asian	Black White	Hispanic White	Asian White
Segregation	0.100	0.331 ***	−0.193	0.029	0.150 **	–0.210 ***
	(0.096)	**(0.123)**	(0.140)	(0.043)	**(0.059)**	**(0.068)**
Gini Coeff.	2.235 ***	2.101 ***	2.258 ***	2.263***	2.244 ***	2.207 ***
	(0.198)	**(0.204)**	(0.196)	(0.196)	(0.195)	(0.195)
Proportion Foreign Born	−0.044	−0.051	−0.052	−0.049	−0.217 **	**0.142**
	(0.085)	(0.084)	(0.084)	(0.085)	**(0.107)**	**(0.104)**
Proportion Female-Headed Household	−1.556 ***	−1.355 ***	−1.631 ***	−1.619 ***	−1.239 **	−1.275 **
	(0.505)	**(0.508)**	(0.506)	(0.510)	**(0.519)**	(0.510)
Proportion Residing in Same Household	−0.426 *	**−0.342**	−0.547 **	−0.443 *	**−0.333**	−0.633 ***
	(0.229)	**(0.226)**	(0.224)	(0.232)	**(0.228)**	(0.223)
Median Age	0.006*	0.005*	0.006 *	0.006*	0.008 **	**0.003**
	(0.003)	(0.003)	(0.003)	(0.003)	**(0.003)**	**(0.003)**
SES	−0.004	−0.003	0.006	−0.003	**−0.014**	−0.003
	(0.011)	(0.010)	(0.012)	(0.011)	**(0.011)**	(0.010)
Home Owner	−0.177	**−0.140**	−0.227 *	−0.195	−0.201	−0.238 *
	(0.139)	**(0.136)**	(0.134)	(0.138)	(0.133)	**(0.133)**
Southern	0.007	0.014	0.014	0.007	0.004	**0.020**
	(0.033)	(0.032)	(0.032)	(0.034)	(0.032)	**(0.032)**
Western	0.058*	0.064**	0.053*	0.058*	**0.046**	0.076**
	(0.030)	**(0.030)**	(0.030)	(0.030)	**(0.030)**	(0.031)
Mid Western	0.053	0.060 *	0.060*	0.055*	0.065 **	0.069**
	(0.033)	(0.032)	(0.033)	(0.033)	**(0.033)**	(0.033)
Total Population (logged)	0.089 ***	0.082***	0.100 ***	0.093 ***	0.088***	0.103***
	(0.014)	**(0.013)**	(0.013)	(0.013)	**(0.013)**	**(0.013)**
Primary City	−0.031	−0.033 *	**−0.028**	−0.029	−0.026	−0.023
	(0.019)	(0.019)	**(0.019)**	(0.019)	(0.019)	(0.019)
Constant	−0.504 *	−0.541 **	−0.528 *	−0.535 **	−0.728 ***	−0.596 **
	(0.274)	(0.270)	(0.271)	(0.272)	(0.280)	(0.270)
Adjusted R2	0.453	0.460	0.454	0.452	0.459	0.462

Standardized coefficients presented; *** *p* < 0.001, ** *p* < 0.01, * *p* < 0.05; Bolded coefficients indicate coefficient was significantly different from Model 1 and 4 (*p* < 0.05), respectively.
